# Sodium Dichloroacetate Stimulates Angiogenesis by Improving Endothelial Precursor Cell Function in an AKT/GSK-3β/Nrf2 Dependent Pathway in Vascular Dementia Rats

**DOI:** 10.3389/fphar.2019.00523

**Published:** 2019-05-17

**Authors:** Hui Zhao, Junqin Mao, Yuan Yuan, Jingjing Feng, Hao Cheng, Guorong Fan, Yuefan Zhang, Tiejun Li

**Affiliations:** ^1^Department of Pharmacy, Punan Hospital, Shanghai, China; ^2^College of Pharmacology, Anhui University of Chinese Medicine, Hefei, China; ^3^Department of Pharmacy, Shanghai Pudong New Area People’s Hospital, Shanghai, China; ^4^Department of Clinical Pharmacy, Shanghai General Hospital, Shanghai JiaoTong University School of Medicine, Shanghai, China; ^5^Department of Pharmacology, College of Pharmacy, Second Military Medical University, Shanghai, China

**Keywords:** sodium dichloroacetate, endothelial progenitor cells, reactive oxygen species, angiogenesis, vascular dementia

## Abstract

Sodium dichloroacetate (DCA) is a mitochondrial pyruvate dehydrogenase kinase inhibitor, and has been shown to display vasoprotective effects in chronic ischemic stroke. The purpose of this study was to evaluate the therapeutic effect of DCA on vascular dementia (VD) and endothelial progenitor cell (EPC)-mediated angiogenesis. After cerebral ischemia-reperfusion in rats, DCA was administered continuously for 21 days; following which, histological analysis, and cognitive functional tests were conducted. Rat bone marrow-derived EPCs were isolated, their function and quantity were measured, and the effects of long-term administration of DCA on EPCs in a rat model of VD was studied. We found that long-term DCA administration improved cognitive function in VD rats, reduced brain infarct size and brain atrophy, increased VEGF and bFGF levels *in vivo*, promoted angiogenesis in damaged areas, and significantly improved EPC function in VD rats. Compared with the VD group, AKT, Nrf2, eNOS expression, and intracellular NO levels were elevated in EPCs of DCA-treated VD rats. In addition, GSK3β and intracellular ROS levels were decreased. Simultaneously, it was found that DCA directly acted on EPCs, and improved EPC functional behavior. Taken together, these findings suggested that long-term DCA administration improved cognitive function in a rat model of VD, and did so in part, by improving EPC function. Observations suggest that prolonged DCA administration might be beneficial in treating VD.

## Introduction

Vascular dementia (VD) is a very frequently seen form of dementia that is recognized as a neurocognitive disorder, caused by stroke and other types of cerebrovascular disorders, which accounts for 15 percent of all dementias seen at the clinic. The risk of VD increases exponentially with age (O’Brien and Thomas, 2015). In fact, VD caused by cerebral vascular injury or vascular circulatory injury might lead to mental disability (Yew et al., 2017), including apathy, depression, agitation, and delusion, which are prevalent and gradually worsen over time (Jin et al., 2015).

A growing body of evidence suggests that following tissue ischemia or vascular endothelial injury, blood vessels can be repaired by bone marrow-derived endothelial progenitor cells (EPCs). EPCs contribute to the formation and recovery of new blood vessels, and are mobilized from the bone marrow to the blood circulation, and can reach sites of vascular injury, at which time they can proliferate and differentiate into endothelial cells to repair damaged blood vessels (Williamson et al., 2012). Recent reports indicate that EPCs encouraged therapeutic effects on long-term stroke prognosis in mice (Fan et al., 2010), and do so by enhancing EPCs function (migration, adhesion, and tube formation), and play protective roles in ischemic stroke (Xin et al., 2016). This suggests that improving EPC function might have therapeutic utility in treating brain damage caused by stroke.

Sodium dichloroacetate (DCA) is a mitochondrial pyruvate dehydrogenase kinase inhibitor, and is an orally absorbable small molecular compound for MELAS syndrome, and children with congenital lactic acidosis and other diseases that are treated in the clinic (Michelakis et al., 2008, 2010). Recent studies had found that DCA acts as a potential vasoprotective agent by inhibiting PDK2 and reducing coronary endarterium proliferation (Deuse et al., 2014). Further, DCA promotes brain regeneration after cerebral ischemia (Hong et al., 2018), which indicates that DCA might play an important role in VD.

Thus, the aim of this study was to validate the hypothesis that DCA could improve cognitive function by promoting EPC function and angiogenesis in VD rats.

## Materials and Methods

### Animals

Adult male SD (Sprague-Dawley) rats (220–230 g) were purchased from the Changzhou CAVENS Laboratory Animal Co., Ltd (Changzhou, China). Food and water were available *ad libitum* under constant temperature (23 ± 2°C) and controlled light conditions (12 h light/dark cycle).

This study was carried out in accordance with the recommendations of “The Care and Use of Laboratory Animals, the Animal Committee of the Second Military Medical University.” The protocol was approved by the “Animal Committee of the Second Military Medical University.” All works were done to prevent animal suffering and to minimize the number of animals used in keeping with the 3R’s principles of animal research.

### Reagents

DCA was purchased from Sigma-Aldrich (St. Louis, MO, United States). Primary antibodies against eNOS (1: 1000), AKT (1: 1000), GSK-3β (1: 1000), and Nrf2 (1:1000) were purchased from Abcam (Cambridge, MA, United States).

### Vascular Dementia (VD) Model

Cerebral ischemia-reperfusion was induced by middle cerebral artery occlusion (MCAO), resulting in a similar pathology to VD (Li et al., 2013). Left middle cerebral arterial occlusion was performed as previously described (Zhou et al., 2010; Zhang et al., 2012). Concisely, anesthesia was induced with 2 percent sodium pentobarbital (50 mg/kg, i.p.). Surgery exposed the left common carotid artery, which isolated, and permitted ligation of the external carotid artery (ECA) with 4-0 silk thread. After an ECA incision, a 4-0 single monofilament coated with poly L-lysine (Beijing Cinontech Co. Ltd, China) was gently introduced (18 ± 20 mm) into the internal carotid artery (ICA), thereby occluding the middle cerebral artery (MCA) origin. After 120 min, the thread was removed to allow for reperfusion, following which, rats were placed in a thermostat at 37 ± 0.5°C prior to recovery. Sham rats were undergone the exact same procedure without arterial ligation and monofilament insertion.

### Experimental Groups and Drug Treatment

Rat behavior was examined at 24 h after reperfusion, and animals showing contralateral forelimb dysfunction were included for further experimentation (Longa et al., 1989). A total of 40 MCAO rats were randomly separated into five groups by lottery drawing: (1) sham group (sham, *N* = 8); (2) VD group (treated with 0.9%NaCl, *N* = 8); (3) 50 mg/kg DCA group (VD treated with 50 mg/kg/day DCA, *N* = 8); (4) 100 mg/kg DCA group (VD treated with 100 mg/kg/day DCA, *N* = 8); and (5) 200 mg/kg DCA group (VD treated with 200 mg/kg/day DCA, *N* = 8). DCA and normal saline were orally administered (by gastric gavage) for 21 consecutive days. After 21 days, the Morris water maze test was performed, and rat bone marrow-derived EPCs were isolated and cultured. In addition, pathological changes and angiogenesis in damaged parts of the brain were assessed.

### Morris Water Maze (MWM)

Morris water maze experiments were performed based on experimental references and appropriate improvements (Morris, 1984; Cantarella et al., 2015). The Morris water maze (MWM) consisted of a black tank filled with black water (Black food additive) (24–25°C), which was divided into four quadrants (I, II, III, and IV). Spatial cues were presented in each quadrant, and a transparent platform with a diameter of 10 cm in the middle of the four-quadrant area was placed with 1–2 cm below the water surface. The MWM study consisted of two parts: (1) positioning navigation test and (2) space exploration test. The experimenter conducted this study without having any knowledge of animal grouping.

In the positioning navigation test, rats started from a random quadrant (except number four-quadrant), and recorded the time required to reach the hidden platform (latency). The maximum time of rats finding the platform was 60 s, Once the rats reached the platform within 60 s and stayed in place for 5 s on the platform, the timer automatically stopped, and allowed the rat to stay for 10 s. If the platform could not be found, then those failed rats were manually moved to the platform and allowed to stay there for 10 s. Each rat received three 1-min training session over a 5 days period.

The space exploration test was conducted on the second day after completing the training test. Briefly, the platform was removed and a quadrant was randomly selected as the starting position. Each rat had 60 s of free swimming to find the original location of the platform. During the procedure, the time was recorded for the time of each rat to locate the target quadrant. After each trial, rats were dried off in their housing facilities with an electric heater for 30 min.

### 2,3,5-Triphenyltetrazolium Chloride (TTC) Staining

After the MCAO procedure was conducted in the rat model, rats were sacrificed (*n* = 5 per group) on the 3rd, 7th, 14th, and 21st day, and the brain were taken for further study. Brains were rinsed with saline to remove excess water, and immediately placed in the freezer for 15 min. The brain tissue was coronally cut into 6 pieces, placed in a 1 percent TTC (Sigma, MO, United States) solution and keepped in an oven at 37°C for 30 min, and fixed in 4 percent paraformaldehyde after staining. According to the order of brain slices, they were arranged vertically and photographed separately. The degree of brain atrophy was calculated using ImageJ software (National Institutes of Health, Bethesda, MD, United States).

### Immunohistochemical Assessment

The rats were euthanized and the brains were fixed with 4 percent paraformaldehyde, and embeddedin paraffin. After conventional slicing, immunostaining with a CD31 antibody (Abcam, Cambridge, MA, United States) was used to identify brain angiogenesis of VD rats.

### Determination of Serum VEGF and bFGF

Quantification of VEGF and bFGF in serum was performed using an ELISA kit (R&D Systems, Minneapolis, MN, United States) according to the manufacturer’s instructions. Measurements were made using a microplate reader (Multiskan MK3; Thermo Fisher Scientific, Inc.).

### Quantification of Peripheral Blood EPCs

Twenty-one days after MCAO surgery, EPCs in the peripheral blood were quantified by flow cytometry (*n* = 5 per group) (Patel et al., 2017; Bianconi et al., 2018). Briefly, rat peripheral blood was obtained and peripheral blood mononuclear cells (PB-MNC) were isolated by Histopaque-10831 (Sigma, MO, United States) density gradient centrifugation. The red blood cells were lysed with ammonium chloride solution, washed twice with PBS, and then incubated with 5 percent bovine serum albumin (BSA, Sigma, MO, United States) for 15 min to block non-specific binding, FITC-VEGFR2 (Abcam, Cambridge, United Kingdom) and PE-labeled CD34 (Abcam, Cambridge, United Kingdom) primary antibody stained cells were incubated for 1 h. The same fluorescein-labeled isotypic-matched I gG was used as a control to determine each stained negative population. Cells were analyzed by flow cytometry (BD Biosciences, CA, United States).

### Bone Marrow-Derived EPCs Culture (BM-EPCs)

Bone marrow-derived EPCs were isolated, and cultured *in vitro* according to previously reported techniques (Dai et al., 2017; Yang J.X. et al., 2018). Briefly, bone marrow mononuclear cells (MNCs) were isolated from rat femurs by density gradient centrifugation over Histopaque-10831 (Sigma, MO, United States). Isolated cells were suspended in endothelial growth medium-2 (EGM-2; Lonza, Basel, Switzerland) with 10 percent fetal bovine serum (FBS), and seeded into a 6-well culture plate that was pre-coated with rat vitronectin (1 μg/mL, Sigma, MO, United States) at a cell density of 5 × 10^6^ cells /well. After 1 day of culture, the non-adherent cells were removed, and the EGM-2 medium was replaced every 3 days. Cells that emerged after 7 days of culture were defined as early EPCs, and used for research (including cell identification, functional assays, and Western blot analysis).

### Characterization of Bone Marrow-Derived MNCs

After 7 days of culture in EGM-2 media, MNCs were washed once with PBS to remove non-adherent MNCs, and incubated with 5 μg/mL Acetylated DiI lipoprotein (Dil-Ac-LDL, Thermo, MA, United States) at 37°C, and 5 percent CO_2_ for 4 h, then washed three times with PBS and fixed with 2 percent paraformaldehyde for 10 min, and finally incubated with 10 μg/mL FITC-labeled Ulex europaeus lectin-1 (FITC-UEA-1, Sigma, MO, United States) at 37°C, 5 percent CO_2_ for 1 h, and before being observed by laser confocal microscopy.

### Flow Cytometry Analysis

Cells were incubated with 5 percent bovine serum albumin (BSA, Sigma, MO, United States) for 15 min to block non-specific binding, following which, cells were stained with FITC-VEGFR2 (Abcam, Cambridge, United Kingdom) for 1 h, and another group of cells was stained with PE-labeled CD34 (Abcam, Cambridge, United Kingdom) primary antibody for 1 h. The same fluorescein-labeled isotypic-matched IgG was used as a control to determine each stained negative population. Cells were analyzed with a flow cytometer (BD Biosciences, CA, United States) (Rehman et al., 2003; Bianconi et al., 2018).

### Migration Assay

Endothelial progenitor cell migration ability was investigated by a modified Boyden chamber assay. EPCs were treated with DCA (200 μM) and bFGF (50 ng/ml) for 48 h. EPCs were cultured with EGM-2 media in a Boyden chamber at adensity of 5 × 10^4^ cells. The lower chamber was filled with EBM-2 supplemented with vascular endothelial growth factor (VEGF, 50 ng/mL). After cells were allowed to migrate for 24 h (37°C, 5% CO_2_), the EPCs were stained with Hochest 33258 (Sigma, MO, United States) for 15 min and fixed in 2% paraformaldehyde. The number of adherent cells were randomly counted in three fields (magnification × 100) of each sample, and the average of each sample was determined (Xin et al., 2016; Peng et al., 2018).

### Cell Adhesion Assay

Approximately 1 × 10^4^ EPCs were uniformly plated in a 96-well plate that was pre-coated with rat vitronectin (1 μg/mL) and incubated at 37°C in 5% CO_2_ for 2 h. PBS was used to wash away non-adherent cells, and EPCs were stained with Hoechst 33258 for 15 min. The number of adherent cells was randomly counted in three fields (magnification × 100) of each sample, and the average numbers of adherent cells in each sample was determined (Peng et al., 2018).

### Tube Formation Assay

Approximately 5 × 10^4^ EPCs were seeded into a 96-well plate coated with Matrigel (80 μL, BD Bioscience, CA, United States) and incubated at 37°C in 5% CO_2_ for 8 h. Tube formation was randomly counted in three fields of view (magnification × 100) for each sample, and the average numbers of tube forming cells in each sample were counted (Yang J.X. et al., 2018).

### Intracellular ROS and NO Measurement

Intracellular ROS levels were determined using dihydroethyl ingot (DHE) (Beyotime, China) staining (Xin et al., 2016; Peng et al., 2018). After 7 days of culture, EPCs from each group were collected and incubated with DHE (10^−6^ mol/ L) for 30 min in the dark. The EPCs were washed twice with PBS, and the fluorescence intensity was analyzed by flow cytometry.

Intracellular NO levels were assayed with membrane-permeable 3-amino,4- aminomethyl-2’,7’-difluorescein, diacetate (DAF-FM DA) (Beyotime, China). After 7 days of culture, EPCs from each group were collected and incubated with DAF-FM DA (10^−6^mol/L) for 30 min in the dark. The EPCs were washed twice with PBS, and the fluorescence intensity was analyzed by flow cytometry.

### Western Blot Assay

Western blot was performed as previously described (Zhu et al., 2016). The EPCs were collected, and the total cellular protein was extracted by a lysate kit (Beyotime Biotechnology, Shanghai, China) according to the manufacturer’s instructions. After determination of protein concentrations by a bicinchoninic acid (BCA) protein assay kit (Pierce, Thermo Fisher Scientific, MA, United States), the total proteins were separated on an 8% SDS-PAGE gel and transferred to nitrocellulose blotting membranes. The membranes were blocked with Tris–buffered saline with 5% non-fat milk for 1 h, and incubated with primary antibody eNOS, AKT, p-AKT, GSK-3β, Nrf2 (1:1000) β-actin (1:3000) (Abcam, Cambridge, United Kingdom) overnight at 4°C, and then washed three times with wash buffer, and incubated with the secondary antibodies for 1 h at room temperature. The strips were visualized by Odyssey Imager with Odyssey 1.1 software (Li-Cor) and quantified using the NIH Image J 1.49p software.

### Statistical Analysis

All data are presented as mean ± SD. Statistical analysis was performed using SPSS version 13.0 software with one or two-way ANOVA, followed by *post hoc* multiple comparisons analysis by the Scheffe test. An alpha value of *P* < 0.05 was considered statistically significant.

## Results

### Sodium Dichloroacetate Improves Cognitive Function of Vascular Dementia and Attenuates Histopathological Characteristics in VD Rats

**FIGURE 1:**
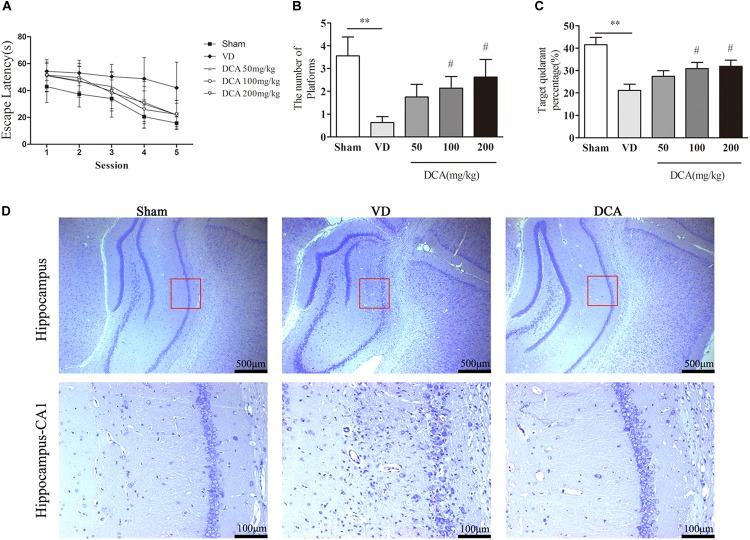
Sodium dichloroacetate (DCA) improves cognitive function of vascular dementia (VD) and attenuates histopathological Characteristics in rats. Morris water maze test procedure determined the spatial learning ability in SD rats, 21 days after MCAO surgery. The escape latency of the learning trajectory, and the percentage time spent in the target quadrant and the number of passes through the platform in the spatial exploration test were calculated. **(A)** The escape latency of the learning trajectory. **(B)** The percentage time spent in the target quadrant (the position of the platform during training). **(C)** The number of passes through the platform within 60 s of commencing the test. ^∗∗^*P* < 0.01 vs. Sham, ^#^*P* < 0.05 vs. VD (*n* = 7–8). **(D)** Nissl staining of the rat hippocampus. Scale bar: 500 μm (up); 100 μm (below). Analyses were statistically tested by two-way ANOVA, followed by *post hoc* multiple comparisons analysis by the Scheffe test.

We assessed the improvement of DCA treatment in rescuing the memory impairment in VD rats using the MWM test, and learning ability by measuring the escape latency (Morris, 1984). During the training course, the escape latency was increased in the VD group as compared with sham rats (*P* < 0.05; Figure 1A). Interestingly, the escape latency of the DCA group was significantly shortened as compared with the VD model group, especially in the 100 and 200 mg/kg DCA treatment groups (*P* < 0.01). At 24 h after the final trial, the space exploration test was performed without the platform, and recording the time spent in the target quadrant and the number of crossings of the platform. Rats in the VD model group displayed shorter periods of time spent in the target quadrant and fewer times crossing the platform compared with rats in the sham group (*P* < 0.01; Figure 1B,C); however, it was longer after treatment with DCA. Rats treated with 100 and 200 mg/kg DCA had significantly increased times that were spent in the target phase quadrant and across the platform as compared with rats in the VD model group (*P* < 0.05); however, there was no significant difference seen in the 50 mg/kg DCA and VD groups (*P* > 0.05; Figure 1B,C).

We further assessed the histopathological changes in VD rats. Nissl staining examined histological changes in the hippocampal CA1 region (Figure 1D). In the sham group, the neurons were in a normal form with clear Nissl bodies and no signs of interstitial edema. In contrast, a large number of degenerated and necrotic neurons were observed in the VD model group, accompanied by nuclear pyknosis, nuclear rupture and dissolution, and interstitial edema. In addition, the number of degenerated and necrotic neurons was reduced in DCA treated rats. Observation indicated that DCA treatment decreased hippocampal damage in VD rats.

**FIGURE 2:**
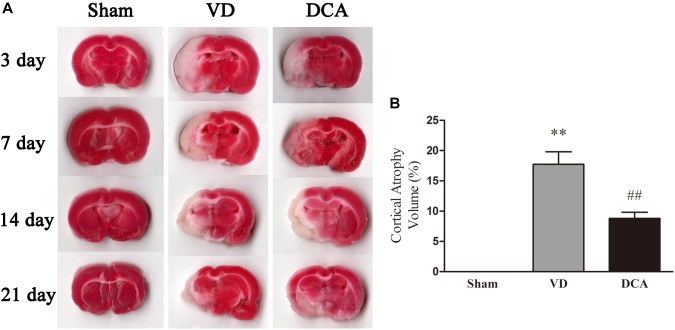
DCA treatment reduces cerebral cortical atrophy in VD rats. TTC staining was used to detect cerebral cortex atrophy in rats, and the degree of brain atrophy in each group was compared. **(A)** Rat brain TTC staining on the 3rd, 7th, 14th, and 21st day (*n* = 5). **(B)** The degree of cerebral cortex atrophy in rats at 21st day. ^∗∗^*P* < 0.01 vs. Sham, ^##^*P* < 0.01 vs. VD (*n* = 5). Analyses were statistically tested by one-way ANOVA.

The rat atrophy area was detected by TTC staining (Figure 2A). The whole brain tissue of the sham treated group was red, with no infarct area or evidence of brain atrophy. Both the VD and the DCA groups formed obvious focal white infarct areas, and showed different degrees of brain atrophy after the seventh day. However, the atrophy size of the DCA group was small, and the degree of brain atrophy was significantly reduced in twenty-one days as compared with the model group (*P* < 0.01, Figure 2B). These observations indicated that DCA can alleviate cerebral infarction and brain atrophy caused by MCAO.

### DCA Treatment Increases Angiogenesis in the Cortex of VD Rats

**FIGURE 3:**
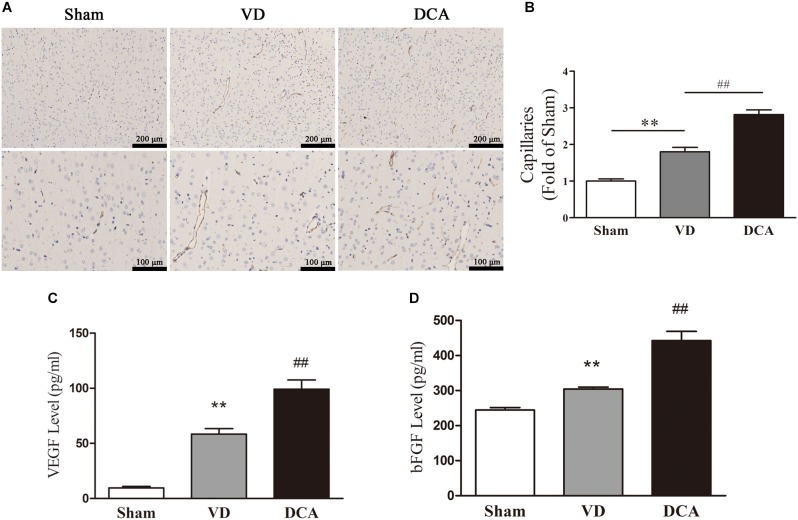
DCA treatment increases angiogenesis in the cortex of VD rats. Immunohistochemistry was used to detect angiogenesis in the rat brain, and ELISA was used to detect the levels of VEGF and bFGF in the serum. **(A)** CD31 immunostaining of cerebral microvessels in rats. **(B)** The bar graph of the number of microvessels in the rats. ^∗∗^*P* < 0.01 vs. Sham,^##^*P* < 0.01 vs. VD (*n* = 5). Scale bar: 100 μm (up), 50 μm (below). **(C)** Levels of VEGF in rat serum. **(D)** The levels of bFGF in rat serum. ^∗∗^*P* < 0.01 vs. Sham, ^##^*P* < 0.01 vs. VD (*n* = 6). Analyses were statistically tested by one-way ANOVA.

Immunohistochemistry was used to examine angiogenesis of the damaged area in the cortex of VD rats (Figure 3A). The capillary density of DCA-treated VD rats was significantly increased as compared with the VD group (*P* < 0.01; Figure 3B), which indicates that DCA promoted angiogenesis in the cortex of the rat brain.

We further examined the levels of VEGF and bFGF in rat serum to elucidate the mechanism by which DCA promotes angiogenesis. As shown in Figure 3C,D, serum levels of VEGF and bFGF were elevated in the VD model group as compared with the sham group (*P* < 0.01). Interestingly, DCA (100 mg/kg) treatment significantly increased VEGF and bFGF levels as compared with the VD group (*P* < 0.01).

### Characterization of EPCs

MNCs that were isolated from the rat bone marrow were seeded in vitronectin-coated six-well plates. Three days after seeding, central round cells and elongated peripheral cells appeared (Supplementary Figure 1A). By 7 days, spindle-shaped adherent EPCs were observed (Supplementary Figure 1A). After 14 days of culture, endothelial-like EPCs with a pebbled morphological character were found (Supplementary Figure 1A). EPCs also stained positive with DiI-acLDL and UEA-1 (Supplementary Figure 1B). Flow cytometry demonstrated that EPCs highly expressed both CD34 and VEGFR2 (Supplementary Figure 1C). These characteristics are consistent with those previously reported in EPCs (Dai et al., 2017).

### DCA Promotes BM-EPC Function

**FIGURE 4:**
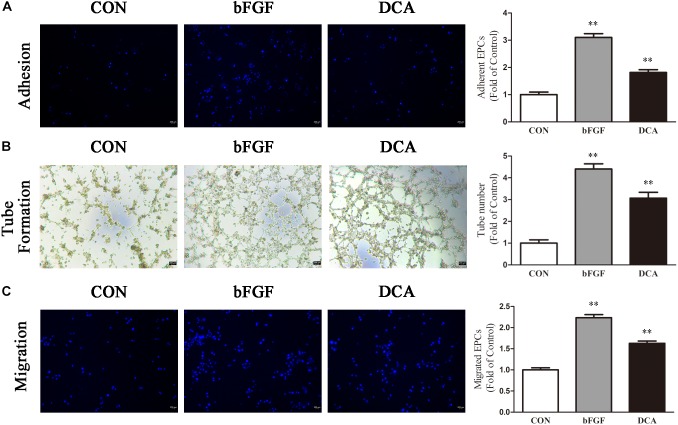
DCA enhances BM-EPC functions. EPC function in each group was compared. **(A)** Adhesion assay for BM-EPC by staining with Hoechst 33258. **(B)** Tube formation of BM-EPC. **(C)** EPCs were cultured in EGM-2 media using transwell chambers at a density of 5 × 10^4^ cells. After migration was permitted for 24 h, the EPCs were stained with Hochest 33258. Migration assay for BM-EPC was determined by counting the number of adherent cells. ^∗∗^*P* < 0.01 vs. Control (*n* = 4). Scale bar: 200 μm. Analyses were statistically tested by one-way ANOVA.

For cell adhesion experiments, results showed that DCA (200 mM) treated cells were more potent than control cells (*p* < 0.01; Figure 4A), and bFGF was used as the positive control. Furthermore, tube forming ability was evaluated through an *in vitro* angiogenesis assay. As shown in Figure 4B, after incubation on Matrigel for 6 h, tubule numbers in the DCA-treated groups were significantly increased as compared with controls (*P* < 0.01). Thus, DCA potently enhanced the *in vitro* tube forming ability of BM-EPCs. Transwell experiments were used to investigate cell migration that was influenced by DCA (200 mM). When treated with DCA, BM-EPCs showed enhanced migration (Figure 4C), and increased numbers of migrating BM-EPCs were found as compared with controls (*p* < 0.01). In general, DCA enhances BM-EPC functionality.

### DCA Increases Peripheral Blood EPC Numbers and Enhances Their Function in VD Rats

**FIGURE 5:**
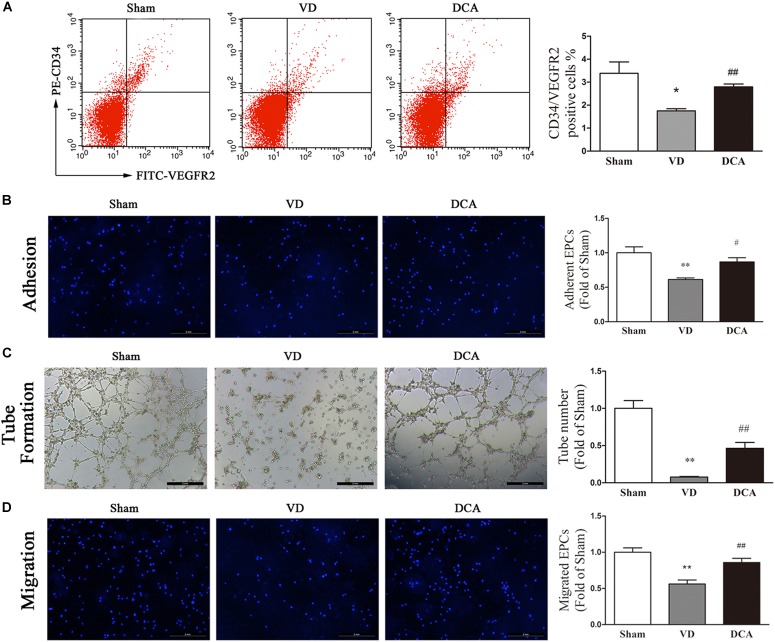
DCA increases peripheral blood endothelial progenitor cells (EPCs) and enhances BM-EPC function in VD rats. **(A)** The number of EPCs in the peripheral blood was detected by flow cytometry (*n* = 5–6). **(B)** EPC function in each group was compared. EPCs were uniformly seeded in culture plates that were pre-coated with rat vitronectin. Adhesion assay for BM-EPC by staining with Hoechst 33258. **(C)** Tube formation of BM-EPC. **(D)** EPCs were cultured in EGM-2 media using transwell chambers at a density of 5 × 10^4^ cells. After migration was permitted for 24 h, the EPCs were stained with Hochest 33258. Migration assay for BM-EPC was determined by counting the number of adherent cells. ^∗^*P* < 0.05, ^∗∗^*P* < 0.01 vs. Sham. ^#^*P* < 0.05, ^##^*P* < 0.01 vs. VD (*n* = 4). Scale bar: 200 μm. Analyses were statistically tested by one-way ANOVA.

After DCA administration for 21 days, the number of peripheral blood EPCs and the functional activity of BM-EPCs in rats were measured. The number of peripheral blood CD34^+^/VEGFR2^+^ EPCs was significantly lower in the VD group as compared with the sham group (*P* < 0.05). Interestingly, DCA administration for 21 days significantly increased the number of peripheral blood CD34^+^/VEGFR2^+^ EPCs (*P* < 0.01; Figure 5A).

Bone marrow-derived EPCs culture adhesion ability decreased in VD rats (*P* < 0.01). By contrast, the adhesion of BM-EPCs in DCA-treated VD rats increased significantly (*P* < 0.05; Figure 5B). BM-EPC tube-forming ability of VD rats was decreased (*P* < 0.01), while the tube-forming ability of BM-EPCs was significantly increased in DCA-treated VD rats (*P* < 0.01; Figure 5C). BM-EPC migration was analyzed by the transwell chamber assay, which showed that BM-EPC migration ability was decreased in VD rats (*P* < 0.01). By contrast, the migration ability of BM-EPCs in DCA-treated VD rats increased significantly (*P* < 0.01; Figure 5D). In general, DCA enhanced endothelial progenitor function in VD rats.

### DCA Treatment Increased NO Levels and Decreased ROS Levels in BM-EPCs

**FIGURE 6:**
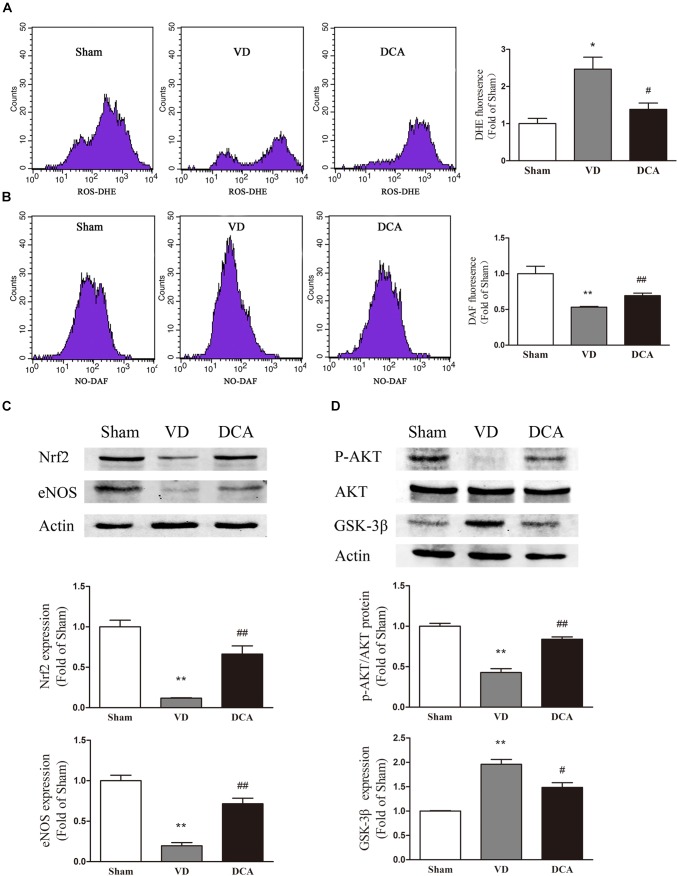
DCA increased NO levels and decreased ROS levels in BM-EPC. Flow cytometry was used to detect the intracellular levels of ROS **(A)** and NO **(B)** in BM-EPCs, *n* = 3. Western Blot assay was used to detect the protein expression of Nrf2 and eNOS **(C)** and P-Akt, eNOS, and GSK-3β **(D)** in BM-EPCs (*n* = 3). ^∗^*P* < 0.05, ^∗∗^*P* < 0.01 vs. Sham. ^#^*P* < 0.05, ^##^*P* < 0.01 vs. VD. Analyses were statistically tested by one-way ANOVA.

To determine the underlying mechanism of DCA in EPC protection, we evaluated intracellular ROS and NO levels in rat BM-EPCs (Figure 6A,B). Intracellular NO levels were significantly lower in BM-EPCs of VD rats as compared with sham controls (*P* < 0.01), while ROS levels were comparatively higher (*P* < 0.05). However, in the DCA-treated group, the NO levels in EPCs of DCA rats were significantly increased (*P* < 0.01), and the ROS levels were decreased (*P* < 0.05) as compared with the VD group. The results showed that DCA treatment increased NO levels and reduced levels of ROS in EPCs of VD rats.

### DCA Improves EPC Function Through an AKT/GSK-3β/Nrf2-Dependent Pathway

We wished to further research the molecular mechanism of VD-induced increases in ROS synthesis by EPCs. We evaluated the expression of AKT, GSK-3β, Nrf2, and eNOS in EPCs. By Western blot analysis, the protein expression of GSK-3β in the VD group was significantly increased compared with controls (*P* < 0.01 and *P* < 0.05, respectively), while the protein expression levels of AKT, Nrf2, and eNOS were comparatively decreased as compared with the control group (*P* < 0.05). In the DCA-treated group, GSK-3β protein expression was significantly decreased as compared with the VD group (*P* < 0.01 and *P* < 0.05, respectively; Figure 6D). Protein expression levels of AKT, Nrf2, and eNOS were all significantly increased (*P* < 0.05; Figure 6C,D). These results indicated that the AKT/GSK-3β signaling pathways are involved in improved EPC function following treatment with DCA.

## Discussion

Previous studies have shown that DCA plays an essential role in vascular protection and in promoting revascularization of blood vessels (Deuse et al., 2014; Hong et al., 2018) and improves vascular calcification in patients with atherosclerosis (Yang Y. et al., 2018). DCA might also have therapeutic effects in the context of vascular-related diseases.

In the current study, we first evaluated the effect of DCA on VD rats. The cognitive ability of rats tested by the MWM procedure found that an improvement was clearly seen in the cognitive ability of VD rats at 100 mg/kg/day DCA administration, and this dose of DCA could improve the cognitive function in this model and reduce brain damage and brain atrophy in VD rats. The hippocampus is highly sensitive to brain damage, and hippocampal lesions are one of the main causes of dementia (De Leon et al., 1989). The hippocampal lesions in the CA1 area more likely cause dementia with deficits in memory. Reducing lesions in this area of the brain is conducive to improving cognitive function (Ihara et al., 2018). Thus, improved cognitive function following DCA treatment can be partly attributed to reduced hippocampal damage. In this study, Nissl staining of the brain in VD rats showed that the integrity of hippocampal formation was impaired and the number of necrotic neurons was also increased. DCA treatment significantly increased the number of Nissl bodies and reduced neuronal necrosis. This indicates that DCA has therapeutic effects on VD rats. However, its molecular mechanism needs further exploration.

We found that DCA administration promoted the levels of VEGF and bFGF in the serum of VD rats to increase. VEGF is a potential angiogenic promoter that stimulates the recruitment, proliferation and differentiation of endothelial cells and increases vascular permeability through the VEGF signaling pathway. In addition, VEGF enhances endothelial PDGF-B expression, whereas bFGF, as a crucial vascular growth factor can enhance neural PDGF receptor-β (PDGFR-β) expression. The synergistic effect of VEGF and bFGF promotes mature blood vessel formation and mitosis (Kano et al., 2005; Dashnyam et al., 2017), indicating that the proangiogenic effects of DCA might be related to the release of these cytokines.

DCA treatment significantly improves EPC function (i.e., migration, adhesion, and tube formation) in VD rats. EPC play a vital role in ischemic vascular repair and angiogenesis, which had been previously shown to be a new target cell for the treatment of vascular-related diseases (Asahara et al., 1997). Recent reports have shown that increasing the function of BM- EPCs promotes wound healing and wound neovascularization in diabetic mice (Zhuge et al., 2018). Studies have shown that peripheral blood EPC levels, as biomarkers of vascular function, and homing to vascular injury sites in response to ischemic stimuli, promotes the restoration of endothelial integrity of damaged blood vessels and actively participates in neovascularization (Asahara et al., 1999; Li et al., 2012). Although the number of circulating EPCs peaked within 7 days after ischemic stroke, it was found that ischemic injury resulted in a gradual decrease in the number of circulating EPCs (Martí-Fàbregas et al., 2015), which is insufficient to promote vascular remodeling and regeneration, and an increasement in the number of circulating EPCs was associated with an improved recovery of brain function, and decreased infarct growth (Sobrino et al., 2007). Thus, it is important to improve the number of circulating EPCs after stroke to recover brain function.

We found that DCA treatment significantly increased circulating EPCs levels in VD rats. Consistent with DCA-enhanced EPC functions, DCA intervention also significantly increased local angiogenesis in the rat cerebral cortex. We found that DCA improved the function of EPCs in VD rats. Thus, in order to further verify whether DCA acts directly on EPCs *in vivo* to play a role in the treatment of VD, we performed *in vitro* experiments with DCA, to directly interfere with EPCs. The results showed that DCA significantly improved EPC function.

Several studies have shown that protein kinases including AKT, mTOR and eNOS kinase, regulate angiogenesis (Saraswati et al., 2013), and that the AKT /eNOS pathway is generally considered to be the most important pathway. The expression of Akt and endothelial nitric oxide synthase (eNOS) is thought to enhance EPC and endothelial cell migration and angiogenesis (Huang et al., 2018). GSK3β is a ubiquitously expressed serine/threonine protein kinase (Ma et al., 2010). In EPCs, small molecule inhibition of GSK3β increases the function of EPCs, and does so by increasing angiogenesis in the ischemic model and by improving arterial vascular injury after the repair process has been signaled (Werner et al., 2003). GSK3β expression was elevated in BM-EPCs that were harvested from VD rats, and GSK-3β was significantly reduced in BM-EPC in DCA rats. Studies have shown that decreased expression of eNOS and decreased levels of intracellular NO might be associated with EPC dysfunction (Marrotte et al., 2010), and increased production of ROS in EPCs might provoke apoptosis (Cui et al., 2015).

Nrf2 is a key redox sensor and one of the major regulators of antioxidant response. Nrf2 binds to regulatory antioxidant elements and activates transcription of many antioxidant genes (e.g., HO-1, NQO-1, etc.) to counter ROS accumulation and the potential for subsequent ROS-mediated cellular and DNA damage (Murakami, 2015). In the present study, we found that the expression of Nrf2 was significantly reduced in EPCs of VD rats and that the expression of Nrf2 was elevated in EPCs after DCA treatment. This result indicates that the antioxidant capacity of EPCs can be improved by activating Nrf2 expression. The important role of Nrf2 in endothelial function has also been widely recognized. Florczyk et al. (2014) reported that the lack of Nrf2 attenuates endothelial cell survival, proliferation and angiogenesis both *in vitro* and *in vivo*. Vascular endothelial growth factor increases the nuclear localization of Nrf2 and the expression of its target genes HO-1 and NQO-1, which stimulates the lumen formation of endothelial cells. Our studies in EPCs are consistent with these findings in endothelial cells, and these findings demonstrate that up-regulation of Nrf2 functional expression plays an important role in enhancing EPC function.

In the present study, we observed a significant increasement in AKT, eNOS, and Nrf2 expression in DCA-treated VD rat EPCs, and an increasement in intracellular NO levels with concordant decreases in intracellular ROS. Thus, increasing NO bioavailability by reducing GSK3β expression and increasing both eNOS and Nrf2 expression might partially contribute to enhanced EPC function following DCA treatment.

**FIGURE 7:**
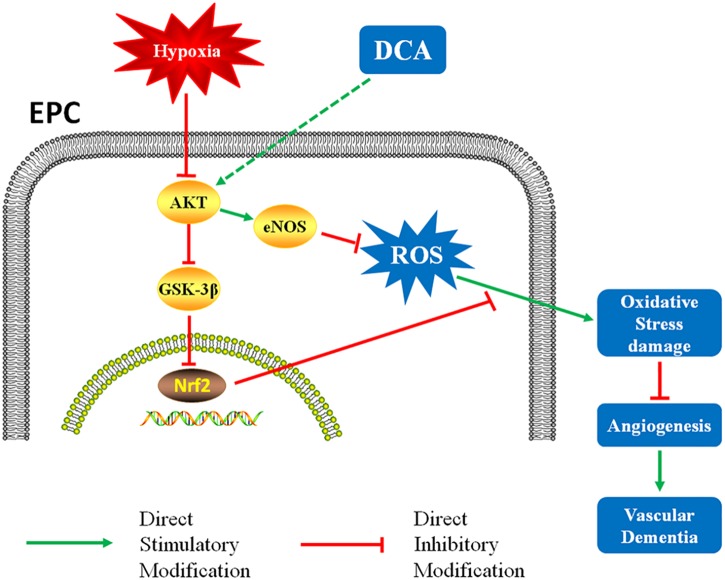
DCA might reduces oxidative stress damage through the AKT/Nrf2 pathway and enhances EPCs function, resulting in faster vessel formation in rats.

In conclusion, DCA intervention significantly improved EPC function, increased angiogenesis in the injured area, and improved cognitive function in a rat model of VD (Figure 7). These results serve as a new finding for the use of DCA in the setting of VD – providing a potential treatment for patients with vascular-related diseases.

This study demonstrates for the first time that DCA can improve cognitive and EPC functions in a rat model of VD.

## Ethics Statement

This study was carried out in accordance with the recommendations of “the Care and Use of Laboratory Animals, Animal Committee of the Second Military Medical University.” The protocol was approved by “the Animal Committee of the Second Military Medical University.”

## Author Contributions

TL and YZ conceived and designed the study. YY, JF, and HC constructed the animal model. HZ and JM performed the cell experiments and Western Blot experiment, analyzed the data, and wrote the manuscript. TL, GF, and YZ revised the manuscript.

## Conflict of Interest Statement

The authors declare that the research was conducted in the absence of any commercial or financial relationships that could be construed as a potential conflict of interest.
