# Supplementary material for: Sodium Dichloroacetate Stimulates Angiogenesis by Improving Endothelial Precursor Cell Function in an AKT/GSK-3β/Nrf2 Dependent Pathway in Vascular Dementia Rats
Source: Front Pharmacol. 2019 May 17;10:523. doi: 10.3389/fphar.2019.00523 (PMC6533549; doi:10.3389/fphar.2019.00523)
Supplement: Supplementary file 1 [file Image_1.pdf]

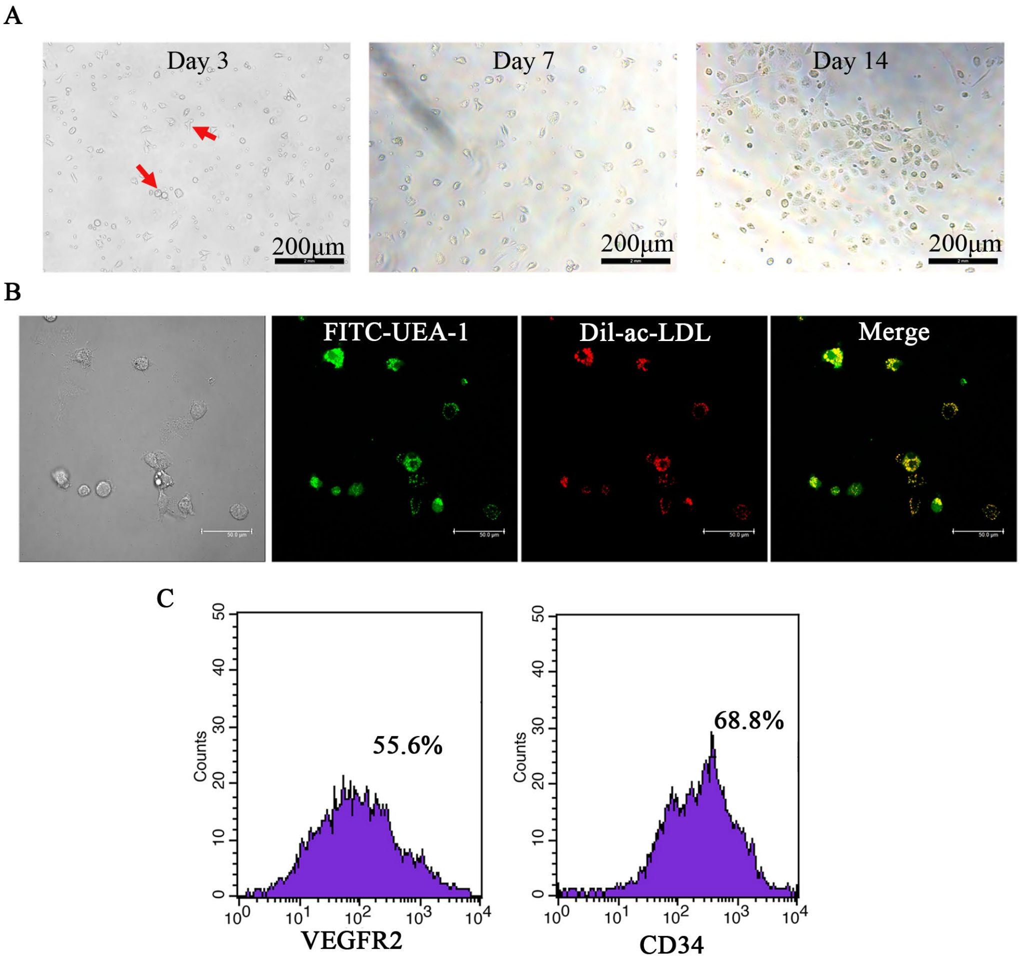

Supplementary FIGURE 1 | Immunofluorescence identification and immunophenotyping of bone marrow derived-EPCs.
Bone marrow derived-EPCs were isolated from rat femurs, and Dil-Ac-LDL and FITC-UEA-1 were added. **(A)** Morphology of bone marrow derived-EPCs cultures at different time after plating. Scale bar: 200μm. **(B)** Dil-acLDL and FITC-UEA-1 staining. Scale bar: 50μm. **(C)** Histograms for cell surface markers by flow cytometry. Three independent experiments were performed.
